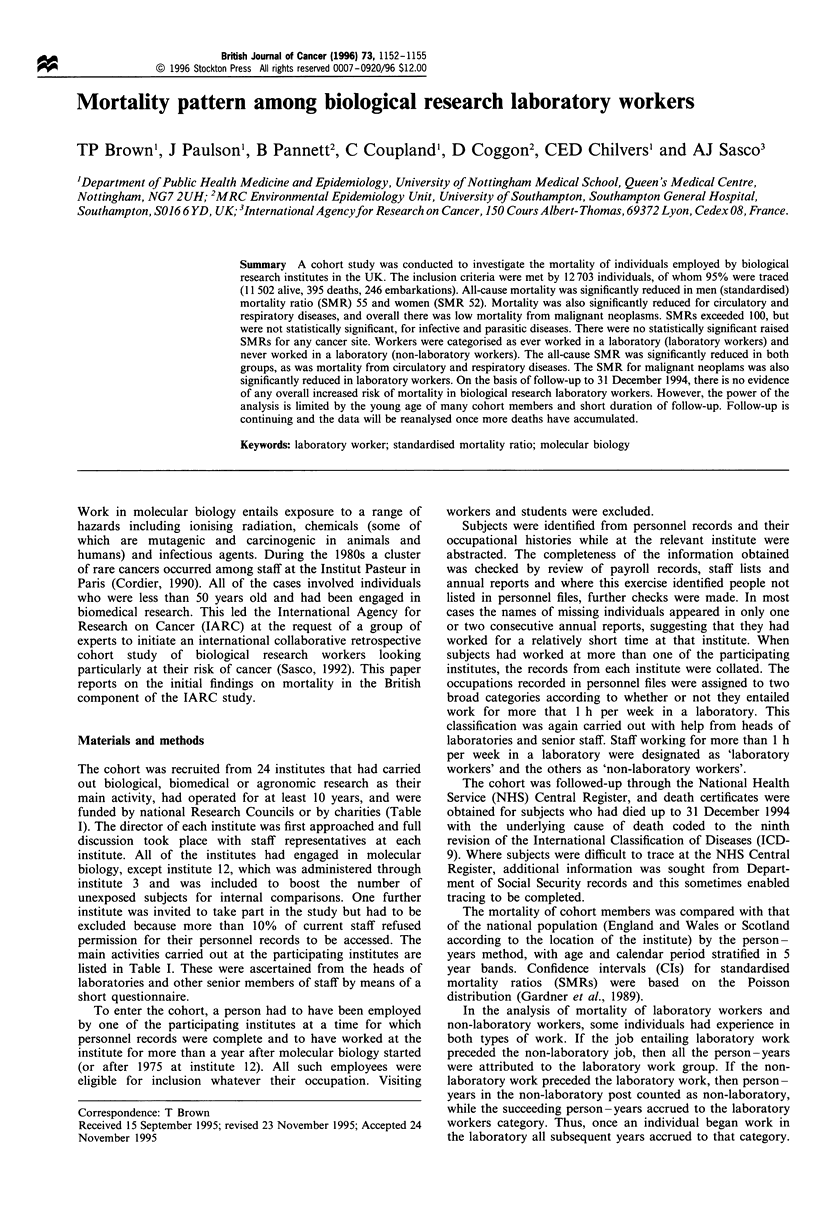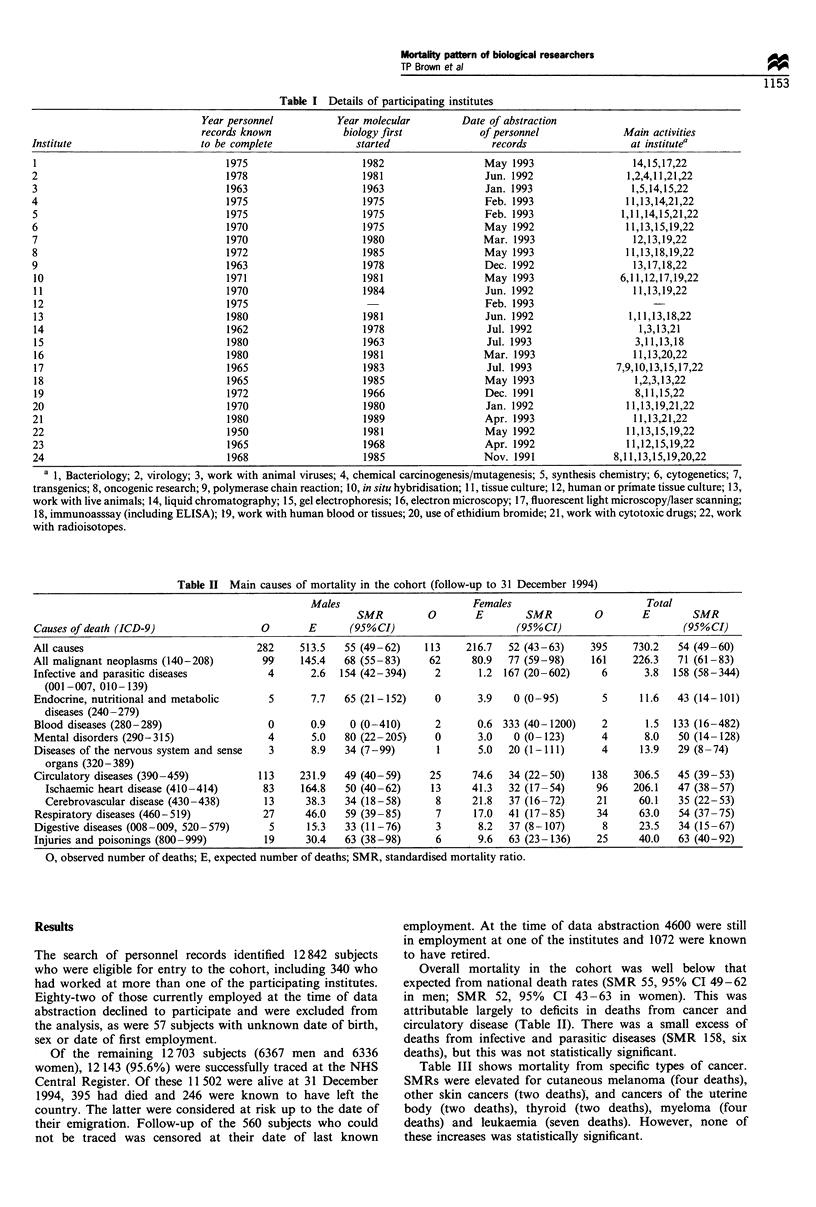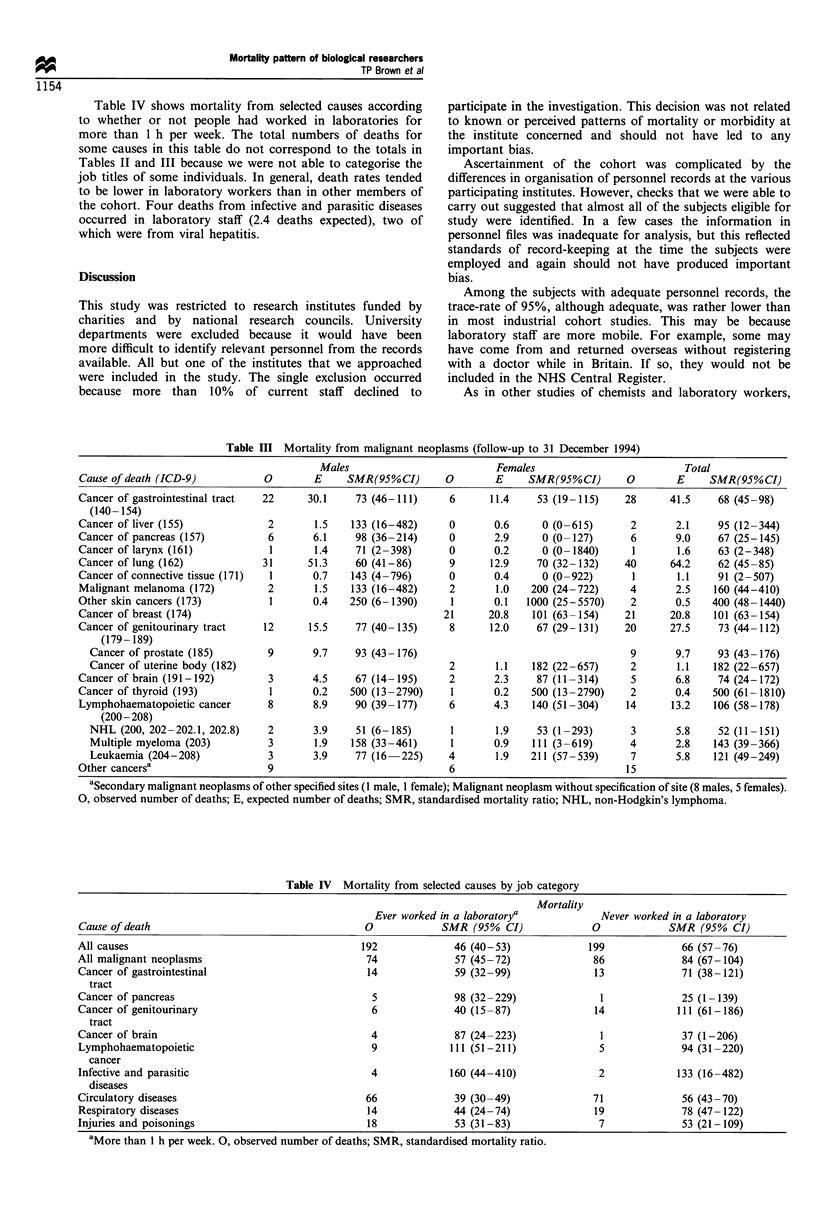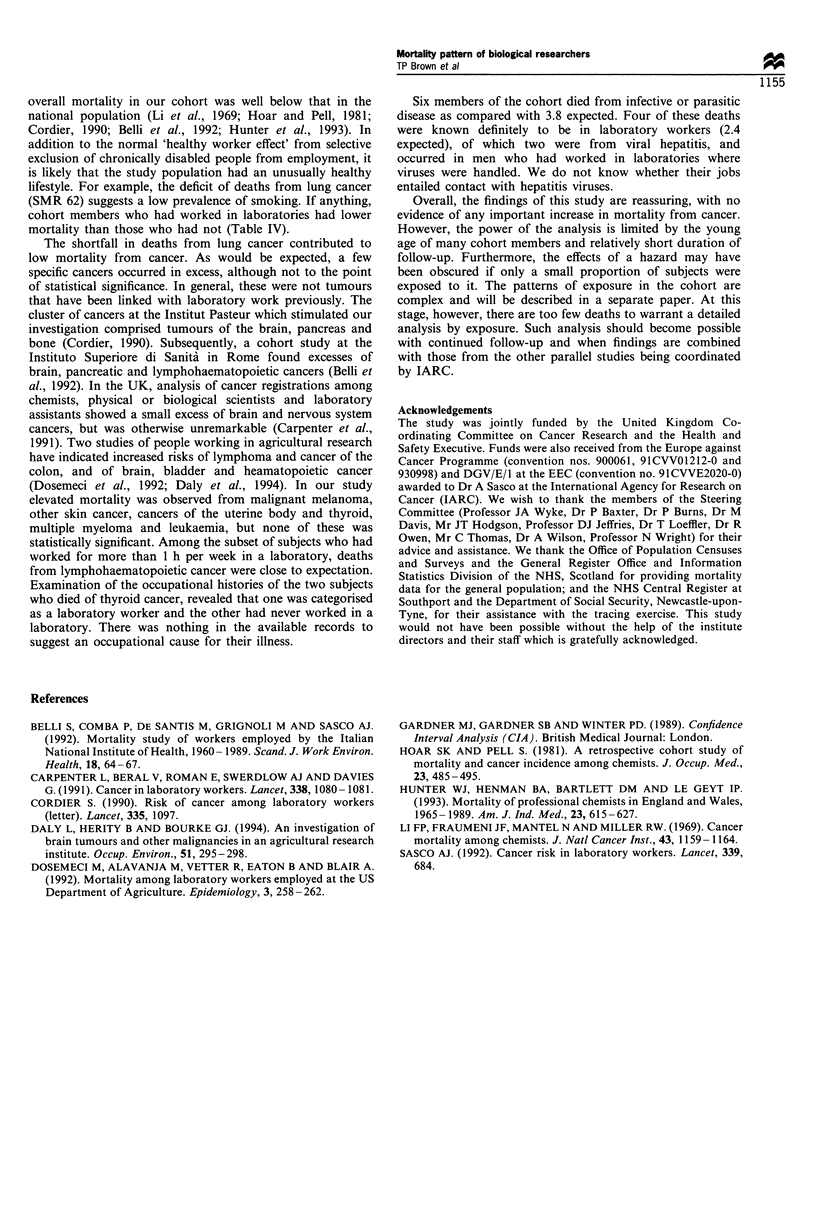# Mortality pattern among biological research laboratory workers.

**DOI:** 10.1038/bjc.1996.221

**Published:** 1996-05

**Authors:** T. P. Brown, J. Paulson, B. Pannett, C. Coupland, D. Coggon, C. E. Chilvers, A. J. Sasco

**Affiliations:** Department of Public Health Medicine and Epidemiology, University of Nottingham Medical School, Queen's Medical Centre, UK.

## Abstract

A cohort study was conducted to investigate the mortality of individuals employed by biological research institutes in the UK. The inclusion criteria were met by 12,703 individuals, of whom 95% were traced (11,502 alive, 395 deaths, 246 embarkations). All-cause mortality was significantly reduced in men (standardised) mortality ratio (SMR) 55 and women (SMR 52). Mortality was also significantly reduced for circulatory and respiratory diseases, and overall there was low mortality from malignant neoplasms. SMRs exceeded 100, but were not statistically significant, for infective and parasitic diseases. There were no statistically significant raised SMRs for any cancer site. Workers were categorised as ever worked in a laboratory (laboratory workers) and never worked in a laboratory (non-laboratory workers). The all-cause SMR was significantly reduced in both groups, as was mortality from circulatory and respiratory diseases. The SMR for malignant neoplams was also significantly reduced in laboratory workers. On the basis of follow-up to 31 December 1994, there is no evidence of any overall increased risk of mortality in biological research laboratory workers. However, the power of the analysis is limited by the young age of many cohort members and short duration of follow-up. Follow-up is continuing and the data will be reanalysed once more deaths have accumulated.


British Journal of Cancer (1996) 73, 1152-1155
© 1996 Stockton Press All rights reserved 0007-0920/96 $12.00

### Mortality pattern among biological research laboratory workers

TP Brown[1], J Paulson[1], B Pannett[2], C Coupland[1], D Coggon[2], CED Chilvers[1] and AJ Sasco[3]

*[1]Department of Public Health Medicine and Epidemiology, University of Nottingham Medical School, Queen's Medical Centre, Nottingham, NG7 2UH; [2]MRC Environmental Epidemiology Unit, University of Southampton, Southampton General Hospital, Southampton, S016 6YD, UK; [3]International Agency for Research on Cancer, 150 Cours Albert-Thomas, 69372 Lyon, Cedex 08, France.*

**Summary** A cohort study was conducted to investigate the mortality of individuals employed by biological research institutes in the UK. The inclusion criteria were met by 12 703 individuals, of whom 95% were traced (11 502 alive, 395 deaths, 246 embarkations). All-cause mortality was significantly reduced in men (standardised) mortality ratio (SMR) 55 and women (SMR 52). Mortality was also significantly reduced for circulatory and respiratory diseases, and overall there was low mortality from malignant neoplasms. SMRs exceeded 100, but were not statistically significant, for infective and parasitic diseases. There were no statistically significant raised SMRs for any cancer site. Workers were categorised as ever worked in a laboratory (laboratory workers) and never worked in a laboratory (non-laboratory workers). The all-cause SMR was significantly reduced in both groups, as was mortality from circulatory and respiratory diseases. The SMR for malignant neoplams was also significantly reduced in laboratory workers. On the basis of follow-up to 31 December 1994, there is no evidence of any overall increased risk of mortality in biological research laboratory workers. However, the power of the analysis is limited by the young age of many cohort members and short duration of follow-up. Follow-up is continuing and the data will be reanalysed once more deaths have accumulated.

**Keywords:** laboratory worker; standardised mortality ratio; molecular biology

Work in molecular biology entails exposure to a range of hazards including ionising radiation, chemicals (some of which are mutagenic and carcinogenic in animals and humans) and infectious agents. During the 1980s a cluster of rare cancers occurred among staff at the Institut Pasteur in Paris (Cordier, 1990). All of the cases involved individuals who were less than 50 years old and had been engaged in biomedical research. This led the International Agency for Research on Cancer (IARC) at the request of a group of experts to initiate an international collaborative retrospective cohort study of biological research workers looking particularly at their risk of cancer (Sasco, 1992). This paper reports on the initial findings on mortality in the British component of the IARC study.

### Materials and methods

The cohort was recruited from 24 institutes that had carried out biological, biomedical or agronomic research as their main activity, had operated for at least 10 years, and were funded by national Research Councils or by charities (Table I). The director of each institute was first approached and full discussion took place with staff representatives at each institute. All of the institutes had engaged in molecular biology, except institute 12, which was administered through institute 3 and was included to boost the number of unexposed subjects for internal comparisons. One further institute was invited to take part in the study but had to be excluded because more than 10% of current staff refused permission for their personnel records to be accessed. The main activities carried out at the participating institutes are listed in Table I. These were ascertained from the heads of laboratories and other senior members of staff by means of a short questionnaire.

To enter the cohort, a person had to have been employed by one of the participating institutes at a time for which personnel records were complete and to have worked at the institute for more than a year after molecular biology started (or after 1975 at institute 12). All such employees were eligible for inclusion whatever their occupation. Visiting workers and students were excluded.

Subjects were identified from personnel records and their occupational histories while at the relevant institute were abstracted. The completeness of the information obtained was checked by review of payroll records, staff lists and annual reports and where this exercise identified people not listed in personnel files, further checks were made. In most cases the names of missing individuals appeared in only one or two consecutive annual reports, suggesting that they had worked for a relatively short time at that institute. When subjects had worked at more than one of the participating institutes, the records from each institute were collated. The occupations recorded in personnel files were assigned to two broad categories according to whether or not they entailed work for more that 1 h per week in a laboratory. This classification was again carried out with help from heads of laboratories and senior staff. Staff working for more than 1 h per week in a laboratory were designated as 'laboratory workers' and the others as 'non-laboratory workers'.

The cohort was followed-up through the National Health Service (NHS) Central Register, and death certificates were obtained for subjects who had died up to 31 December 1994 with the underlying cause of death coded to the ninth revision of the International Classification of Diseases (ICD-9). Where subjects were difficult to trace at the NHS Central Register, additional information was sought from Department of Social Security records and this sometimes enabled tracing to be completed.

The mortality of cohort members was compared with that of the national population (England and Wales or Scotland according to the location of the institute) by the person-years method, with age and calendar period stratified in 5 year bands. Confidence intervals (CIs) for standardised mortality ratios (SMRs) were based on the Poisson distribution (Gardner *et al.*, 1989).

In the analysis of mortality of laboratory workers and non-laboratory workers, some individuals had experience in both types of work. If the job entailing laboratory work preceded the non-laboratory job, then all the person-years were attributed to the laboratory work group. If the non-laboratory work preceded the laboratory work, then person-years in the non-laboratory post counted as non-laboratory, while the succeeding person-years accrued to the laboratory workers category. Thus, once an individual began work in the laboratory all subsequent years accrued to that category.

Correspondence: T Brown
Received 15 September 1995; revised 23 November 1995; Accepted 24 November 1995

**Table I** Details of participating institutes

| *Institute* | *Year personnel records known to be complete* | *Year molecular biology first started* | *Date of abstraction of personnel records* | *Main activities at institute*[a] |
|---|---|---|---|---|
| 1 | 1975 | 1982 | May 1993 | 14,15,17,22 |
| 2 | 1978 | 1981 | Jun. 1992 | 1,2,4,11,21,22 |
| 3 | 1963 | 1963 | Jan. 1993 | 1,5,14,15,22 |
| 4 | 1975 | 1975 | Feb. 1993 | 11,13,14,21,22 |
| 5 | 1975 | 1975 | Feb. 1993 | 1,11,14,15,21,22 |
| 6 | 1970 | 1975 | May 1992 | 11,13,15,19,22 |
| 7 | 1970 | 1980 | Mar. 1993 | 12,13,19,22 |
| 8 | 1972 | 1985 | May 1993 | 11,13,18,19,22 |
| 9 | 1963 | 1978 | Dec. 1992 | 13,17,18,22 |
| 10 | 1971 | 1981 | May 1993 | 6,11,12,17,19,22 |
| 11 | 1970 | 1984 | Jun. 1992 | 11,13,19,22 |
| 12 | 1975 | — | Feb. 1993 | — |
| 13 | 1980 | 1981 | Jun. 1992 | 1,11,13,18,22 |
| 14 | 1962 | 1978 | Jul. 1992 | 1,3,13,21 |
| 15 | 1980 | 1963 | Jul. 1993 | 3,11,13,18 |
| 16 | 1980 | 1981 | Mar. 1993 | 11,13,20,22 |
| 17 | 1965 | 1983 | Jul. 1993 | 7,9,10,13,15,17,22 |
| 18 | 1965 | 1985 | May 1993 | 1,2,3,13,22 |
| 19 | 1972 | 1966 | Dec. 1991 | 8,11,15,22 |
| 20 | 1970 | 1980 | Jan. 1992 | 11,13,19,21,22 |
| 21 | 1980 | 1989 | Apr. 1993 | 11,13,21,22 |
| 22 | 1950 | 1981 | May 1992 | 11,13,15,19,22 |
| 23 | 1965 | 1968 | Apr. 1992 | 11,12,15,19,22 |
| 24 | 1968 | 1985 | Nov. 1991 | 8,11,13,15,19,20,22 |

[a] 1, Bacteriology; 2, virology; 3, work with animal viruses; 4, chemical carcinogenesis/mutagenesis; 5, synthesis chemistry; 6, cytogenetics; 7, transgenics; 8, oncogenic research; 9, polymerase chain reaction; 10, *in situ* hybridisation; 11, tissue culture; 12, human or primate tissue culture; 13, work with live animals; 14, liquid chromatography; 15, gel electrophoresis; 16, electron microscopy; 17, fluorescent light microscopy/laser scanning; 18, immunoasssay (including ELISA); 19, work with human blood or tissues; 20, use of ethidium bromide; 21, work with cytotoxic drugs; 22, work with radioisotopes.

**Table II** Main causes of mortality in the cohort (follow-up to 31 December 1994)

| *Causes of death (ICD-9)* | *Males* O | *Males* E | *Males* SMR (95%CI) | *Females* O | *Females* E | *Females* SMR (95%CI) | *Total* O | *Total* E | *Total* SMR (95%CI) |
|---|---|---|---|---|---|---|---|---|---|
| All causes | 282 | 513.5 | 55 (49–62) | 113 | 216.7 | 52 (43–63) | 395 | 730.2 | 54 (49–60) |
| All malignant neoplasms (140–208) | 99 | 145.4 | 68 (55–83) | 62 | 80.9 | 77 (59–98) | 161 | 226.3 | 71 (61–83) |
| Infective and parasitic diseases (001–007, 010–139) | 4 | 2.6 | 154 (42–394) | 2 | 1.2 | 167 (20–602) | 6 | 3.8 | 158 (58–344) |
| Endocrine, nutritional and metabolic diseases (240–279) | 5 | 7.7 | 65 (21–152) | 0 | 3.9 | 0 (0–95) | 5 | 11.6 | 43 (14–101) |
| Blood diseases (280–289) | 0 | 0.9 | 0 (0–410) | 2 | 0.6 | 333 (40–1200) | 2 | 1.5 | 133 (16–482) |
| Mental disorders (290–315) | 4 | 5.0 | 80 (22–205) | 0 | 3.0 | 0 (0–123) | 4 | 8.0 | 50 (14–128) |
| Diseases of the nervous system and sense organs (320–389) | 3 | 8.9 | 34 (7–99) | 1 | 5.0 | 20 (1–111) | 4 | 13.9 | 29 (8–74) |
| Circulatory diseases (390–459) | 113 | 231.9 | 49 (40–59) | 25 | 74.6 | 34 (22–50) | 138 | 306.5 | 45 (39–53) |
| Ischaemic heart disease (410–414) | 83 | 164.8 | 50 (40–62) | 13 | 41.3 | 32 (17–54) | 96 | 206.1 | 47 (38–57) |
| Cerebrovascular disease (430–438) | 13 | 38.3 | 34 (18–58) | 8 | 21.8 | 37 (16–72) | 21 | 60.1 | 35 (22–53) |
| Respiratory diseases (460–519) | 27 | 46.0 | 59 (39–85) | 7 | 17.0 | 41 (17–85) | 34 | 63.0 | 54 (37–75) |
| Digestive diseases (008–009, 520–579) | 5 | 15.3 | 33 (11–76) | 3 | 8.2 | 37 (8–107) | 8 | 23.5 | 34 (15–67) |
| Injuries and poisonings (800–999) | 19 | 30.4 | 63 (38–98) | 6 | 9.6 | 63 (23–136) | 25 | 40.0 | 63 (40–92) |

O, observed number of deaths; E, expected number of deaths; SMR, standardised mortality ratio.

### Results

The search of personnel records identified 12 842 subjects who were eligible for entry to the cohort, including 340 who had worked at more than one of the participating institutes. Eighty-two of those currently employed at the time of data abstraction declined to participate and were excluded from the analysis, as were 57 subjects with unknown date of birth, sex or date of first employment.

Of the remaining 12 703 subjects (6367 men and 6336 women), 12 143 (95.6%) were successfully traced at the NHS Central Register. Of these 11 502 were alive at 31 December 1994, 395 had died and 246 were known to have left the country. The latter were considered at risk up to the date of their emigration. Follow-up of the 560 subjects who could not be traced was censored at their date of last known employment. At the time of data abstraction 4600 were still in employment at one of the institutes and 1072 were known to have retired.

Overall mortality in the cohort was well below that expected from national death rates (SMR 55, 95% CI 49–62 in men; SMR 52, 95% CI 43–63 in women). This was attributable largely to deficits in deaths from cancer and circulatory disease (Table II). There was a small excess of deaths from infective and parasitic diseases (SMR 158, six deaths), but this was not statistically significant.

Table III shows mortality from specific types of cancer. SMRs were elevated for cutaneous melanoma (four deaths), other skin cancers (two deaths), and cancers of the uterine body (two deaths), thyroid (two deaths), myeloma (four deaths) and leukaemia (seven deaths). However, none of these increases was statistically significant.

Table IV shows mortality from selected causes according to whether or not people had worked in laboratories for more than 1 h per week. The total numbers of deaths for some causes in this table do not correspond to the totals in Tables II and III because we were not able to categorise the job titles of some individuals. In general, death rates tended to be lower in laboratory workers than in other members of the cohort. Four deaths from infective and parasitic diseases occurred in laboratory staff (2.4 deaths expected), two of which were from viral hepatitis.

### Discussion

This study was restricted to research institutes funded by charities and by national research councils. University departments were excluded because it would have been more difficult to identify relevant personnel from the records available. All but one of the institutes that we approached were included in the study. The single exclusion occurred because more than 10% of current staff declined to participate in the investigation. This decision was not related to known or perceived patterns of mortality or morbidity at the institute concerned and should not have led to any important bias.

Ascertainment of the cohort was complicated by the differences in organisation of personnel records at the various participating institutes. However, checks that we were able to carry out suggested that almost all of the subjects eligible for study were identified. In a few cases the information in personnel files was inadequate for analysis, but this reflected standards of record-keeping at the time the subjects were employed and again should not have produced important bias.

Among the subjects with adequate personnel records, the trace-rate of 95%, although adequate, was rather lower than in most industrial cohort studies. This may be because laboratory staff are more mobile. For example, some may have come from and returned overseas without registering with a doctor while in Britain. If so, they would not be included in the NHS Central Register.

As in other studies of chemists and laboratory workers,

**Table III** Mortality from malignant neoplasms (follow-up to 31 December 1994)

| *Cause of death (ICD-9)* | Males *O* | *E* | *SMR(95%CI)* | Females *O* | *E* | *SMR(95%CI)* | Total *O* | *E* | *SMR(95%CI)* |
|---|---|---|---|---|---|---|---|---|---|
| Cancer of gastrointestinal tract (140–154) | 22 | 30.1 | 73 (46–111) | 6 | 11.4 | 53 (19–115) | 28 | 41.5 | 68 (45–98) |
| Cancer of liver (155) | 2 | 1.5 | 133 (16–482) | 0 | 0.6 | 0 (0–615) | 2 | 2.1 | 95 (12–344) |
| Cancer of pancreas (157) | 6 | 6.1 | 98 (36–214) | 0 | 2.9 | 0 (0–127) | 6 | 9.0 | 67 (25–145) |
| Cancer of larynx (161) | 1 | 1.4 | 71 (2–398) | 0 | 0.2 | 0 (0–1840) | 1 | 1.6 | 63 (2–348) |
| Cancer of lung (162) | 31 | 51.3 | 60 (41–86) | 9 | 12.9 | 70 (32–132) | 40 | 64.2 | 62 (45–85) |
| Cancer of connective tissue (171) | 1 | 0.7 | 143 (4–796) | 0 | 0.4 | 0 (0–922) | 1 | 1.1 | 91 (2–507) |
| Malignant melanoma (172) | 2 | 1.5 | 133 (16–482) | 2 | 1.0 | 200 (24–722) | 4 | 2.5 | 160 (44–410) |
| Other skin cancers (173) | 1 | 0.4 | 250 (6–1390) | 1 | 0.1 | 1000 (25–5570) | 2 | 0.5 | 400 (48–1440) |
| Cancer of breast (174) | | | | 21 | 20.8 | 101 (63–154) | 21 | 20.8 | 101 (63–154) |
| Cancer of genitourinary tract (179–189) | 12 | 15.5 | 77 (40–135) | 8 | 12.0 | 67 (29–131) | 20 | 27.5 | 73 (44–112) |
| Cancer of prostate (185) | 9 | 9.7 | 93 (43–176) | | | | 9 | 9.7 | 93 (43–176) |
| Cancer of uterine body (182) | | | | 2 | 1.1 | 182 (22–657) | 2 | 1.1 | 182 (22–657) |
| Cancer of brain (191–192) | 3 | 4.5 | 67 (14–195) | 2 | 2.3 | 87 (11–314) | 5 | 6.8 | 74 (24–172) |
| Cancer of thyroid (193) | 1 | 0.2 | 500 (13–2790) | 1 | 0.2 | 500 (13–2790) | 2 | 0.4 | 500 (61–1810) |
| Lymphohaematopoietic cancer (200–208) | 8 | 8.9 | 90 (39–177) | 6 | 4.3 | 140 (51–304) | 14 | 13.2 | 106 (58–178) |
| NHL (200, 202–202.1, 202.8) | 2 | 3.9 | 51 (6–185) | 1 | 1.9 | 53 (1–293) | 3 | 5.8 | 52 (11–151) |
| Multiple myeloma (203) | 3 | 1.9 | 158 (33–461) | 1 | 0.9 | 111 (3–619) | 4 | 2.8 | 143 (39–366) |
| Leukaemia (204–208) | 3 | 3.9 | 77 (16—225) | 4 | 1.9 | 211 (57–539) | 7 | 5.8 | 121 (49–249) |
| Other cancers[a] | 9 | | | 6 | | | 15 | | |

[a]Secondary malignant neoplasms of other specified sites (1 male, 1 female); Malignant neoplasm without specification of site (8 males, 5 females). O, observed number of deaths; E, expected number of deaths; SMR, standardised mortality ratio; NHL, non-Hodgkin's lymphoma.

**Table IV** Mortality from selected causes by job category

| *Cause of death* | Ever worked in a laboratory[a] *O* | *SMR (95% CI)* | Never worked in a laboratory *O* | *SMR (95% CI)* |
|---|---|---|---|---|
| All causes | 192 | 46 (40–53) | 199 | 66 (57–76) |
| All malignant neoplasms | 74 | 57 (45–72) | 86 | 84 (67–104) |
| Cancer of gastrointestinal tract | 14 | 59 (32–99) | 13 | 71 (38–121) |
| Cancer of pancreas | 5 | 98 (32–229) | 1 | 25 (1–139) |
| Cancer of genitourinary tract | 6 | 40 (15–87) | 14 | 111 (61–186) |
| Cancer of brain | 4 | 87 (24–223) | 1 | 37 (1–206) |
| Lymphohaematopoietic cancer | 9 | 111 (51–211) | 5 | 94 (31–220) |
| Infective and parasitic diseases | 4 | 160 (44–410) | 2 | 133 (16–482) |
| Circulatory diseases | 66 | 39 (30–49) | 71 | 56 (43–70) |
| Respiratory diseases | 14 | 44 (24–74) | 19 | 78 (47–122) |
| Injuries and poisonings | 18 | 53 (31–83) | 7 | 53 (21–109) |

[a]More than 1 h per week. O, observed number of deaths; SMR, standardised mortality ratio.

overall mortality in our cohort was well below that in the national population (Li *et al.*, 1969; Hoar and Pell, 1981; Cordier, 1990; Belli *et al.*, 1992; Hunter *et al.*, 1993). In addition to the normal 'healthy worker effect' from selective exclusion of chronically disabled people from employment, it is likely that the study population had an unusually healthy lifestyle. For example, the deficit of deaths from lung cancer (SMR 62) suggests a low prevalence of smoking. If anything, cohort members who had worked in laboratories had lower mortality than those who had not (Table IV).

The shortfall in deaths from lung cancer contributed to low mortality from cancer. As would be expected, a few specific cancers occurred in excess, although not to the point of statistical significance. In general, these were not tumours that have been linked with laboratory work previously. The cluster of cancers at the Institut Pasteur which stimulated our investigation comprised tumours of the brain, pancreas and bone (Cordier, 1990). Subsequently, a cohort study at the Instituto Superiore di Sanità in Rome found excesses of brain, pancreatic and lymphohaematopoietic cancers (Belli *et al.*, 1992). In the UK, analysis of cancer registrations among chemists, physical or biological scientists and laboratory assistants showed a small excess of brain and nervous system cancers, but was otherwise unremarkable (Carpenter *et al.*, 1991). Two studies of people working in agricultural research have indicated increased risks of lymphoma and cancer of the colon, and of brain, bladder and heamatopoietic cancer (Dosemeci *et al.*, 1992; Daly *et al.*, 1994). In our study elevated mortality was observed from malignant melanoma, other skin cancer, cancers of the uterine body and thyroid, multiple myeloma and leukaemia, but none of these was statistically significant. Among the subset of subjects who had worked for more than 1 h per week in a laboratory, deaths from lymphohaematopoietic cancer were close to expectation. Examination of the occupational histories of the two subjects who died of thyroid cancer, revealed that one was categorised as a laboratory worker and the other had never worked in a laboratory. There was nothing in the available records to suggest an occupational cause for their illness.

Six members of the cohort died from infective or parasitic disease as compared with 3.8 expected. Four of these deaths were known definitely to be in laboratory workers (2.4 expected), of which two were from viral hepatitis, and occurred in men who had worked in laboratories where viruses were handled. We do not know whether their jobs entailed contact with hepatitis viruses.

Overall, the findings of this study are reassuring, with no evidence of any important increase in mortality from cancer. However, the power of the analysis is limited by the young age of many cohort members and relatively short duration of follow-up. Furthermore, the effects of a hazard may have been obscured if only a small proportion of subjects were exposed to it. The patterns of exposure in the cohort are complex and will be described in a separate paper. At this stage, however, there are too few deaths to warrant a detailed analysis by exposure. Such analysis should become possible with continued follow-up and when findings are combined with those from the other parallel studies being coordinated by IARC.

**Acknowledgements**
The study was jointly funded by the United Kingdom Co-ordinating Committee on Cancer Research and the Health and Safety Executive. Funds were also received from the Europe against Cancer Programme (convention nos. 900061, 91CVV01212-0 and 930998) and DGV/E/1 at the EEC (convention no. 91CVVE2020-0) awarded to Dr A Sasco at the International Agency for Research on Cancer (IARC). We wish to thank the members of the Steering Committee (Professor JA Wyke, Dr P Baxter, Dr P Burns, Dr M Davis, Mr JT Hodgson, Professor DJ Jeffries, Dr T Loeffler, Dr R Owen, Mr C Thomas, Dr A Wilson, Professor N Wright) for their advice and assistance. We thank the Office of Population Censuses and Surveys and the General Register Office and Information Statistics Division of the NHS, Scotland for providing mortality data for the general population; and the NHS Central Register at Southport and the Department of Social Security, Newcastle-upon-Tyne, for their assistance with the tracing exercise. This study would not have been possible without the help of the institute directors and their staff which is gratefully acknowledged.

### References

BELLI S, COMBA P, DE SANTIS M, GRIGNOLI M AND SASCO AJ. (1992). Mortality study of workers employed by the Italian National Institute of Health, 1960–1989. *Scand. J. Work Environ. Health*, **18**, 64–67.

CARPENTER L, BERAL V, ROMAN E, SWERDLOW AJ AND DAVIES G. (1991). Cancer in laboratory workers. *Lancet*, **338**, 1080–1081.

CORDIER S. (1990). Risk of cancer among laboratory workers (letter). *Lancet*, **335**, 1097.

DALY L, HERITY B AND BOURKE GJ. (1994). An investigation of brain tumours and other malignancies in an agricultural research institute. *Occup. Environ.*, **51**, 295–298.

DOSEMECI M, ALAVANJA M, VETTER R, EATON B AND BLAIR A. (1992). Mortality among laboratory workers employed at the US Department of Agriculture. *Epidemiology*, **3**, 258–262.

GARDNER MJ, GARDNER SB AND WINTER PD. (1989). *Confidence Interval Analysis (CIA)*. British Medical Journal: London.

HOAR SK AND PELL S. (1981). A retrospective cohort study of mortality and cancer incidence among chemists. *J. Occup. Med.*, **23**, 485–495.

HUNTER WJ, HENMAN BA, BARTLETT DM AND LE GEYT IP. (1993). Mortality of professional chemists in England and Wales, 1965–1989. *Am. J. Ind. Med.*, **23**, 615–627.

LI FP, FRAUMENI JF, MANTEL N AND MILLER RW. (1969). Cancer mortality among chemists. *J. Natl Cancer Inst.*, **43**, 1159–1164.

SASCO AJ. (1992). Cancer risk in laboratory workers. *Lancet*, **339**, 684.